# Esophageal schwannoma: Case report and epidemiological, clinical, surgical and immunopathological analysis^☆^

**DOI:** 10.1016/j.ijscr.2018.10.084

**Published:** 2019-01-10

**Authors:** Luiz Carlos de Araújo Souza, Thiago David Alves Pinto, Hugo Oliveira de Figueiredo Cavalcanti, Alexandre Rezende Rezende, Ana Luiza Alves Nicoletti, Cinthia Mares Leão, Vinícius Carvalhêdo Cunha

**Affiliations:** a Undergraduates of Medicine in the University Center of Brasilia (UniCEUB) and Researchers in the Department of Cytopathology and Pathological Anatomy of the Base Institute of the Federal District (NUCAP-IHBDF), Brasilia, Brazil; b Physician Anatomopathologist of Diagnose laboratory and Cytopathology and Pathological Anatomy of the Base Institute of the Federal District (NUCAP-IHBDF), Brasilia, Brazil; c Resident Physicians in Anatomical Pathology of the Federal District Base Institute (NUCAP-IHBDF), Brasília, Brazil; d Physician Anatomopathologist of Cytopathology and Pathological Anatomy of the Base Institute of the Federal District (NUCAP-IHBDF), Brasilia, Brazil

**Keywords:** Case report, Esophageal schwannoma, S-100 protein, CD 34, CD 117

## Abstract

**Introduction:**

Schwannoma is a tumor of the peripheral nervous system originated in the Schwann cells of the neural sheath.

**Presentation of case:**

A 43-years-old male complained of odynophagia, dysphagia and hemoptysis. The upper gastrointestinal endoscopy showed a smooth elevated lesion, 20 cm from the incisor teeth, occupying the entire lumen of the esophagus. The chest computed tomography (CT) scan showed a lesion of 7 cm and superior mediastinal, lower paraesophageal and cardiac enlarged lymph nodes. A posterolateral thoracotomy was performed with total esophagectomy without intraoperative complications. The anatomopathological analysis revealed fusocellular mesenchymal neoplasia of low malignancy potential. The immunohistochemical study showed positivity for S-100 protein and KI67 antibodies and absence of staining for CD117, CD34, ALK protein, SMA and Desmin. Thus, the morphological and immunohistochemical findings pointed to the diagnosis of esophageal Schwannoma.

**Discussion:**

Although rare and indolent, Schwannoma occurs in the peripheral nervous system, being uncommon in the esophagus.

**Conclusion:**

The immunohistochemical study is essential for the diagnosis, which is based on the positivity for S-100 protein and absence of staining for CD34 and CD117.

## Highlights

• The schwannoma of the esophagus is a rare case report.
• We present a review of the main characteristics of this disease.
• Aspects covered epidemiological, clinical, surgical, histopathological and immunohistochemical.

## 1 Introduction

Schwannoma (neurilemmoma) can be found in any neural segment covered by the neural sheath. Schwann cell tumors of the peripheral nervous system were named Neurilemmoma by Stout and Carson in 1935 [1].

Schwannoma or Neurilemmoma is a tumor of the peripheral nervous system originated in Schwann cells. Commonly, the term “Schwannoma” refers to a benign, slow-growing tumor. The rare cancerous cases should be called malignant Schwannoma of malignant nerve sheath tumor [2]. This type of tumor occurs more frequently in the head and neck, extremities and retroperitoneum. Rarely, it can be found in the gastrointestinal tract [3,4,5,6,7,8,9]. The tumor is part of a group of intramural located neoplasms - gastrointestinal mesenchymal tumors, gastrointestinal stromal tumor (GIST), leiomyoma, leiomyosarcoma and others [4,6,7,9].

The gastrointestinal schwannomas are uncommon and most of them originate in the stomach or intestines. The esophageal schwannomas are extremely rare. In the following study, we report a patient with esophageal schwannoma. Our work was described using the SCARE criteria of guidelines for consensus-based surgical case reports [74].

## 2 Literature review

A search of the terms “schwannoma” and “esophagus” was performed using PubMed (NCBI) database. Only literature between 1989 and 2018 was included. A total of 120 studies were identified and analyzed. The inclusion criteria were studies with epidemiological, clinical, surgical, histopathological and immunohistochemical characteristics described and published in PubMed (NCBI) database. Table 2 contains all the studies that performed at least immunohistochemical test using S-100 protein antibodies for diagnostic analysis. Table 3 contains all the patients with malignant esophageal Schwannoma, regardless of immunohistochemistry for diagnostic analysis. Therefore, for our analysis, the review included 51 studies – 48 studies that performed S-100 protein examination (Table 2) and 3 studies of malignant Schwannoma without immunohistochemical data (Table 3).

The data were arranged and the statistical analysis was performed by the SPSS software version 20.0. In the literature review, we analyzed the epidemiological, clinical, histopathological and immunohistochemical characteristics. In our analysis, patients complaining of discomfort or bad sensation during swallowing and difficulty of swallowing were gathered together and it was considered that they had the same symptom – dysphagia. We collected the information regarding the number of patients per study, sex, age, tumor size (mm), tumor location (cm) from the greater distance of the incisor teeth, clinical data and risk factors reported in the studies (Dysphagia; Dyspnea; Odynophagia; Cough; Hemosputum; Haematemesis; Palpitations; Weight loss; Chest pain; Smoking; Drinking; Back pain; Epigastric pain; Paresthesia of the left hand), surgical data reported in the studies (Surgical Approach; Management) and pathological data concerning immunohistochemistry (Smooth Muscle Actin (SMA); Desmin; CD34; CD117; Protein S-100 (S-100); Vimentin; Neuron-specific enolase (NSE); DOG-1; Protein ALK-1 (ALK-1); Synaptophysin; Chromogranin; Glial fibrillary acidic protein (GFAP); Cytokeratin AE1/AE3 (AE1/AE3).

## 3 Presentation of case

A 43-years-old male visited the hospital with complaints of odynophagia, evolving to sudden onset dysphagia to both solids and liquids, which relieved after hemoptysis. The patient presented a history of arterial hypertension and he reported frequent consumption of alcoholic beverages. An upper gastrointestinal endoscopy showed a smooth elevated lesion, 20 cm from the incisor teeth, preserving the continuity of the mucosa that obstructed all the lumen of the organ.

**Fig. 1:**
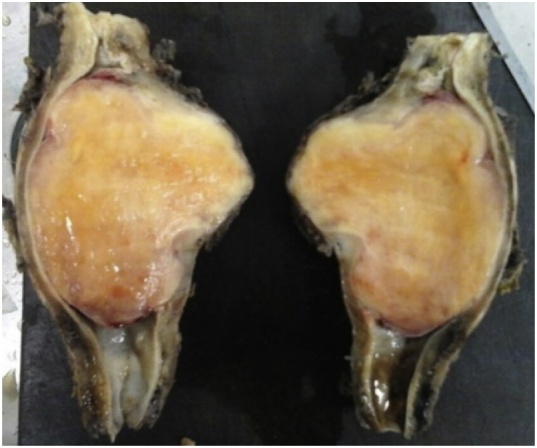
Anatomical parts of the esophageal tumor after right-sided posterolateral thoracotomy with esophagectomy.

A chest computed tomography (CT) scan showed a lesion of 7 cm and superior mediastinal, lower paraesophageal and cardiac enlarged lymph nodes. The patient presented grade I cardiac risk and he was referred to the surgical center. He underwent a thoracotomy via the fifth right intercostal space, with esophagctomy and anisoperistaltic gastric tube preparation. A mass (Fig. 1) was found in the mid-thoracic esophagus and it was sent to anatomopathological study. There were no complications during the procedure (Figures supplemental data 1).

The resected specimen (esophagus segment and celiac trunk lymph node) showed a globose lesion measuring 13.0 × 8.4 × 4.5 cm. The middle third of the segment was poorly delimited and elastic hard, forming a prominence on the outer surface. There was no capsule and it was covered by adventitia. A poorly delimited tumor measuring 7.0 × 7.0 × 4.0 was observed on one side, partially covered by smooth mucosa with erosion foci. That part of the tumor extended to the wall in depth and it was located 4.5 cm from the most distant margin and 1.5 cm from the nearest margin. The cut surface was fasciculated and almost uniformly yellowish-white. Additionally, there was an irregular tissue measuring 3.0 × 2.0 × 1.5 cm, from which celiac trunk lymph nodes were isolated.

Histopathological examination revealed fusocellular mesenchymal neoplasm with low malignancy potential; solid and fasciculate pattern with trabecular areas; localization predominantly in submucosa, permeating own muscle and own lamina; extension of the lesion 7.0 × 7.0 cm; vessels with perivascular hyalinization; moderate intratumoral lymphomononuclear inflammatory infiltrate with lymphoid and peritumoral aggregates; mucosa with epithelial erosion and hemorrhagic foci; necrosis and non-mitotic cells; proximal, radial and distal free surgical margins. The celiac lymph nodes were free of neoplasia.

**Fig. 2:**
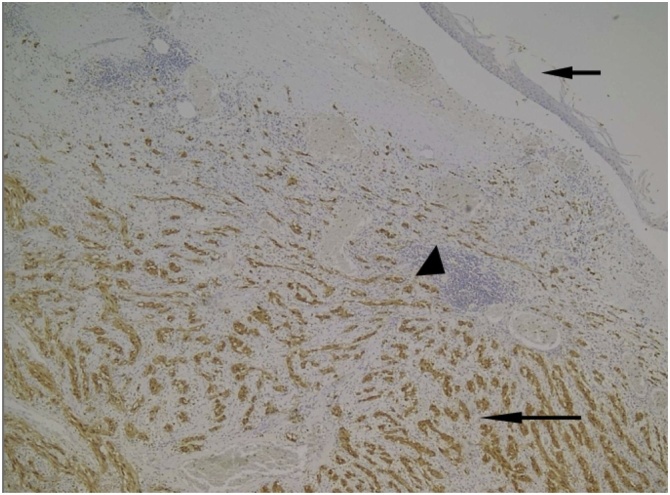
Positive reaction for S-100 in neoplastic cells (**long arrow**) and negative in lymphoid aggregates (**arrowhead**) and squamous epithelium (**short arrow**). (**IHQ 40x**).

Immunohistochemical studies were performed using the following antibodies: SMA (Smooth Muscle Actin), Desmin, CD34, CD117, S-100 protein, ALK protein and KI67. The analysis revealed positivity for the S-100 protein and KI67 and the absence of staining for SMA, Desmin, CD34, CD117 and ALK (Fig. 2 and Table 1). Thus, the morphological and immunohistochemical findings pointed to the diagnosis of esophageal Schwannoma (Figures supplemental data 2–9).

**Table 1:** Immunohistochemical test of our case.

Antibodies		Result
**1)**	Smooth Muscle Actin (SMA)	negative
**2)**	Desmin	negative
**3)**	CD 34	negative
**4)**	CD 117	negative
**5)**	S-100 protein	positive
**6)**	ALK protein	negative
**7)**	KI 67	Positive in 5% of neoplastic cells

**Table 2:** Literature review of epidemiological, clinical, surgical, histopathological and immunohistochemical findings in patients with esophageal Schwannoma [[10], [11], [12], [13], [14], [15], [16], [17], [18], [19], [20], [21], [22], [23], [24], [25], [26], [27], [28], [29], [30], [31], [32], [33], [34], [35], [36], [37], [38], [39], [40], [41], [42], [43], [44], [45], [46], [47], [48], [49], [50], [51], [52], [53], [54], [55], [56], [57]].

[b] (Benign tumor); [m] (Malignant tumor); W (Woman); M (Men); *Greater distance from the incisive teeth; RPT (Right Posterolateral Thoracotomy); VATS (Video-Assisted Thoracoscopic Surgery); RATS (Robot-Assisted Thoracoscopic Surgery); RT (Right Thoracotomy); SE (Subtotal Esophagectomy); E (Enucleation); ER (Endoscopic Removal); TE (Tumor Excision); SMA (Smooth Muscle Actin); S-100 (Protein S-100); NSE (Neuron- Specific Enolase); ALK-1 (Protein ALK-1); GFAP (Glial Fibrillary Acidic Protein); AE1/AE3 (Cytokeratin AE1/AE3).

**Table 3:** Literature review of epidemiological, clinical, surgical, histopathological and immunohistochemical of patients with malignant esophageal Schwannoma.

		Epidemiological data					Clinical Data			Surgical Data		Immunohistochemical Markers								
Author	Year	No. of patients	Sex	Age (year)	Size (mm)	Location (cm)*	Dysphagia	Palpitations	Weight loss	Surgical Approach	Management	SMA	Desmin	CD 34	CD 117	S-100	Vimentin	NSE	Dog-1	KI 67
Iwata et al. [11][m]	1993	1	W	56	48 × 42 × 30					RPT	E					+				
Morita et al. [14][m]	1996	1	W	57	40		+				E					+				
Murase et al. [20][m]	2001	1	W	49	82 × 57 × 37					Right intercostal incision	E	–		–	–	+		+		
Sato N et al. [68][m]	2002	1	M	55	85 × 70 × 40		+				E									
Tsuji et al. [69][m]	2003	1	W	49	82 × 58 × 37		+				E									
Sánchez et al. [26][m]	2004	1	M	54		40	+		+		Total esophagectomy		–		–	+	+			
Kitami et al. [70][m]	2009	1	M	62	37 × 56		+				Chemo-radiation									
Wang et al. [38][m]	2011	1	W	44	55 × 40 × 45	38	+			Thoracotomy	E	–		–	–	+	+			
Mishra et al. [51][m]	2016	1	W	27	12 × 10 × 10	30	+	+	+	Left thoraco-abdominal incision	Esophagectomy	–		–	–	+			–	2-3%

[m] (Malignant tumor); W (Woman); M (Men); *Greater distance from the incisive teeth; RPT (Right posterolateral thoracotomy); E (Enucleation); ER (Endoscopic Removal); TE (Tumor Excision); SMA (Smooth muscle actin); S-100 (Protein S-100); NSE (Neuron- Specific Enolase).

## 4 Results – literature review

After analyzing our case and the 48 studies that performed at least immunohistochemical test using S-100 protein antibodies, we observed that 54 patients were diagnosed with esophageal Schwannoma (Table 2). The patients with a diagnosis of malignant esophageal Schwannoma were grouped and totalled 9 patients in the world. Three studies did not disclose immunohistochemical results (Table 3). The variables analyzed were epidemiological, clinical, surgical, histopathological and immunohistochemical data, which were used to determine statistical conclusions.

After the arrangement of the 54 patients, we analyzed statistically all variables by means of the mean, median, mode, standard deviation and percentage of all patients (Table 4). We observed that 48 (88.9%) patients had benign Schwannoma and 6 (11.1%) patients had malignant Schwannoma; 13 (24.1%) were male and 41 (75.9%) were female; the ages (years) with the highest prevalence (mode) were 39 (5.5%), 57 (5.5%) and 62 (5.5%). The ages varied from 11 to 79 years old. The mean age was 53.72 years-old; median age, 56 years-old and the standard deviation of 13.965 years-old. When analyzing the largest (mm) tumor mass, tumor sizes of 40 (11.3%)mm and 50 (11.3%) were the most prevalent (mode). Tumor sizes ranged from 5 to 150 mm. The mean tumor size was 59.57 mm; median tumor size, 55 mm and the standard deviation of 29.468 mm. When analyzing the tumor location (cm) from the greater distance of the incisor teeth, we found that the most prevalent distance (mode) was 30 (20.0%)cm. Tumor distances varied from 19 to 40 mm. The mean distance was 27.13 cm; median distance, 27.50 cm and standard deviation of 5.476 cm.

**Table 4:** Epidemiological, clinical, surgical, histopathological and immunohistochemical statistical analysis in patients with esophageal Schwannoma with at least S-100 protein labeling.

Analyzed variables		
**Tumor diagnosis:**	**N = 54 (%)**	
[b]/ [m]	48 (88.9)/ 6 (11.1)	
**Sex:**		
[M]/ [W]	13 (24.1)/ 41 (75.9)	
**Age (years):**		
more prevalent (mode)	39 (5.5)/ 57 (5.5)/ 62 (5.5)	
minimum and maximum	11 (1.8)/ 79 (1.8)	
mean/ median/ Std. deviation	53.72/ 56/ 13.965	
**Size (mm) of the largest tumor measure:**	**N = 53 (%)**	
more prevalent (mode)	40 (11.3)/ 50 (11.3)	
minimum and maximum	5 (3.7)/ 150 (1.8)	
mean/ median/ Std. deviation	59.57/ 55/ 29.468	
**Location (cm) longest distance from incisor teeth:**	**N = 30 (%)**	
more prevalent (mode)	30 (20.0)	
minimum and maximum	19 (6.7)/ 40 (3.3)	
mean/ median/ Std. deviation	27.13/ 27.50/ 5.476	
**Symptoms and risk factors reported:**	**N = 67 (%)**	
Dysphagia; Dyspnea; Odynophagia; Cough; Hemosputum; Haematemesis; Palpitations; Weight loss; Chest pain; Smoking; Drinking; Back pain; Epigastric pain; Paresthesia of the left hand.	36 (53.7)/ 7 (10.4)/ 1 (1.4)/ 3 (4.4)/ 2 (2.9)/ 1 (1.4)/ 2 (2.9)/ 3 (4.4)/ 3 (4.4)/ 2 (2.9)/ 3 (4.4)/ 1 (1.4)/ 2 (2.9)/ 1 (1.4)	
**Surgical Approach**	**N = 38 (%)**	
	VATS (26.3)/ RPT (21.0)/ RATS (2.63)	
**Management**	**N = 54 (%)**	
**Immunohistochemical Markers**	**negative (%)**	**positive (%)**
SMA	32 (100)	0 (0.0)
Desmin	22 (100)	0 (0.0)
CD 34	29 (100)	0 (0.0)
CD 117	33 (100)	0 (0.0)
S-100	0 (0.0)	54 (100)
Vimentin	3 (21.4)	11 (78.6)
NSE	0 (0.0)	5 (100)
Dog-1	6 (100)	0 (0.0)
ALK-1	3 (100)	0 (0.0)
Synaptophysin	3 (100)	0 (0.0)
Chromogranin	1 (100)	0 (0.0)
GFAP	1 (33.3)	2 (66.6)
AE1/AE3	2 (100)	0 (0.0)
	**< 5% (%)**	**≥5% (%)**
KI 67	4 (57.1)	3 (42.9)
MIB-1	1 (50.0)	1 (50.0)

[b] (Benign tumor); [m] (Malignant tumor); M (Men); W (Woman); VATS (Video-Assisted Thoracoscopic Surgery); RPT (Right Posterolateral Thoracotomy); RATS (Robot-Assisted Thoracoscopic Surgery); E (Enucleation); SE (Subtotal Esophagectomy); ER (Endoscopic Removal); SMA (Smooth Muscle Actin); S-100 (Protein S-100); NSE (Neuron- Specific Enolase); ALK-1 (Protein ALK-1); GFAP (Glial Fibrillary Acidic Protein); AE1/AE3 (Cytokeratin AE1/AE3).

When analyzing all the symptoms and risk factors of the 54 patients, we observed that dysphagia is reported in 53.7% of clinical complaints, dyspnea in 10.4%, cough/weight loss/chest pain/alcohol consumption in 4.4%, hemoptysis/palpitations/smoking/epigastric pain in 2.9% and odynophagia/hematemesis/back pain/left hand paresthesia in 1.4% of the patients (Table 4). When analyzing which surgical approaches are the most used, we observed that the most performed is the video-assisted thoracoscopic surgery (26.3%), followed by right posterolateral thoracotomy (21.0%) and thoracoscopic surgery assisted by a robot (2.63%). The most used surgical procedure was enucleation (59.2%), followed by subtotal esophagectomy (7.4%) and endoscopic removal (5.5%). When analyzing the immunohistochemical markers, we observed that SMA, Desmin, CD34, CD117, DOG-1, ALK, Synaptophysin, Chromogramin and AE1/AE3 were not registered in all Schwannomas analyzed. The S-100 protein and NSE stained positively in all Schwannomas. Vimentin presented negativity in 3 (21.4%) patients and positivity in 11 (78.6%) patients. GFAP presented negativity in 1 (33.3%) patient and positivity in 2 (66.6%) patients.

## 5 Discussion

**Graph 1:**
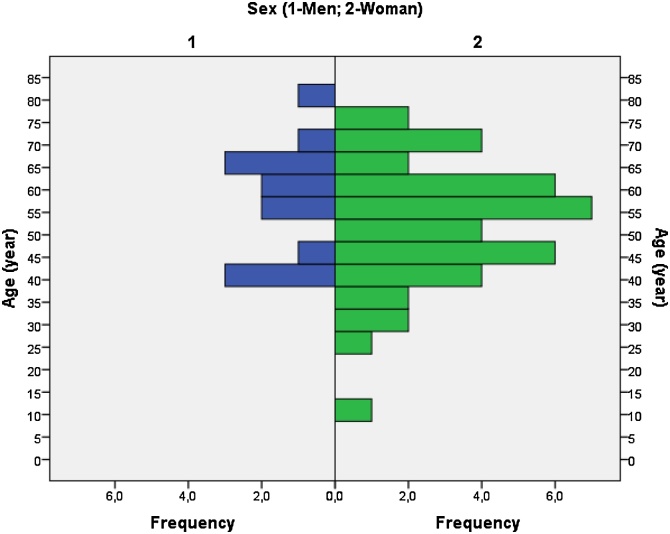
Joint analysis of the Sex (1-Men; 2-Woman) and Age (years) of the 54 patients presented in the literature review.

The Schwannoma represents 0.2–1% of all gastrointestinal tumors and the stomach is the most common site of gastrointestinal schwannoma, while the colon and esophagus are relatively uncommon [3,4,5,6,7,9,58]. The esophageal schwannoma more frequently develops in middle-aged women and is often located the proximal esophagus, with lesion dimensions ranging from 1 to 15 cm [59]. Our literature review has determined that esophagus Schwannoma is more frequent in women (75.9%) and that the higher prevalence age is between the 4th and 6th decades of life (Table 4 and Graph 1).

The Schwannomas are commonly found involving the myelin-forming cells of the 8^th^ cranial nerve, in a condition called vestibular neuroma [60]. Symptoms of this disease depend on the location and include abdominal pain, constipation, bleeding, weight loss or even asymptomatic [4,5,7,9]. In our analysis, the most frequent symptoms were dysphagia (53.7%), dyspnea (10.4%), cough (4.4%), weight loss (4.4%), chest pain (4.4%) and the risk factors the most documented were consumption of alcoholic beverages (4.4%) and smoking (2.9%) (Table 4).

The initial evaluation is made by CT, nuclear magnetic resonance (NMR) and upper gastrointestinal endoscopy to determine the localization, size, density of the lesion and attempt to identify metastasis [4]. The diagnosis of certainty of the mesenchymal tumors is made only with anatomopathological and immunohistochemical study of the surgical specimen [5,6]. In our analysis, the most prevalent size of the tumor is between 40 and 50mmm and the most common location is 30 cm away from the incisor teeth (Table 4).

The Schwannoma is classified as a gastrointestinal mesenchymal tumor, together with GIST, leiomyoma, leiomyosarcoma, desmoid tumor, inflammatory myofibroblastic tumor and others [7]. Schwannomas and neurofibromas correspond histologically to grade I of the WHO classification (benign and encapsulated tumors) [61,62]. Schwannomas are encapsulated and the most cases show two distinct histological patterns, referred to as Antoni A and Antoni B: [61,62,63,64,65]:

• **Antoni A tissue pattern:** the cells are spindle-shaped and compactly arranged. The pattern is characterized by palisades created by the alignment of nuclei that alternate with anucleated, rosy and homogenous or fibrillary zones (Verocay bodies). These bodies consist of cytoplasmic extensions, basement membranes, collagen and reticulin or small groups of fibrils surrounded by lines of palisade-shaped nuclei.
• **Antoni B tissue pattern:** the Antoni B regions contain more loosely arranged. The extensions are not oriented and the nuclei are round, rather to elongated. Occasionally, the cells are starred, creating a resemblance to an astrocytoma. There may be abundant xanthomatous histiocytes. The immunohistochemical and structural features of Antoni B regions suggest that it results from degenerative processes.

In the histological character, the Schwannoma can present significant cellular pleomorphism, lymphoid follicles, rare mitotic figures and rare necrosis points. The fasciculate pattern makes differential diagnosis with GISC, leiomyomas and others. GIST may, on the other hand, have a high mitotic index, foci of necrosis and hemorrhage and there are no lymphoid follicles. Otherwise, leiomyoma does not present mitoses, nor necrosis, hemorrhage or lymphoid follicles [4,7,66].

In genetic issues, there are cytogenetic abnormalities in about half of the schwannomas, regardless of origin. These include loss of material on chromosome 22q, loss of sex chromosome and trisomy of chromosome 7 [61,62]. Most Schwannomas are sporadic, whereas multiple Schwannomas occur in two tumor syndromes: neurofibromatosis type 2 (NF2) and schwannomatosis [61,62]. The pathognomonic finding of NF2 is bilateral schwannoma of the cranial nerve VIII. The schwannomatosis is characterized by multiple peripheral schwannomas in the absence of other signs of NF2 [61,62]. The NF2 gene (on chromosome 22q) and the merlin protein that the gene encodes are implicated in the genesis of about 60% of sporadic schwannomas. In most cases, they are small changes in the reading frame that results in inactivated proteins [61,62]. The loss of merlin expressions, demonstrated by Western blotting or immunohistochemistry, appears to be universal in schwannomas and it is an essential step in its genesis [61,62].

The parameters of schwannoma immunohistochemistry are based on positivity for S-100 protein and on the absence of staining for CD117, CD34, Desmin and specific muscle actin. CD117 and CD34 are positive in GIST and muscle-specific proteins, such as actin, Desmin and caldesmon are positive in smooth muscle tumors such as leiomyoma and leiomyosarcoma. All these proteins are virtually negative in the schwannoma [67]. In our analysis, we observed that the markers SMA, Desmin, CD117, DOG-1, ALK, Synaptophysin, Chromogranin and AE1/AE3 stained negatively in all analyzed schwannomas. The S-100 protein and NSE stained positively in all Schwannomas; Vimentin presented negativity in 3 (21.4%) patients and positivity in 11 (78.6%) patients; GFAP presented negativity in 1 (33.3%) and positivity in 2 (66.6%) patients (Table 4). Therefore, it is evident that GFAP and Vimentin cannot be used as sole markers for the diagnosis of esophageal schwannomas.

The definition of malignant schwannoma is based on a combination of histopathological patterns, such as the presence of mitoses, invasion of muscle layers, cellularity, nuclear atypia and tumor necrosis [26]. Tumor size has also been associated with malignancy. Miettinen et al. [71] suggested that the tumor larger than 6 cm should be considered malignant, although histopathological criteria previously described defining tumor malignancy. After reviewing all malignant Schwannomas in the literature, we found 9 published cases. The malignant schwannomas are more prevalent in females (66.7%); the average age is 50.3 years old; the average tumor size is 57.5 mm; they are located on average 36 cm away from the incisor teeth; the most prevalent symptoms are dysphagia (70.0%), weight loss (20.2%) and palpitations (10.0%); the most used surgical procedure is enucleation of the tumor mass (66.7%); the immunohistochemical markers SMA, Desmin, CD34, CD117, NSE and DOG1 stained negatively on the malignant esophageal schwannoma end S-100 protein, Vimentin and NSE stained positively (Table 3). Therefore, clinical and epidemiological data do not allow the safe inference of an injury with malignant potential.

The therapeutic management of esophageal Schwannoma depends on several factors, such as clinical complaints, tumor size, complications due to tumor growth, pathological data (malignancy, mitotic index and immunohistochemical staining). Thus, most of the studies advocate surgical resection as treatment [19,72]. The enucleation of tumor mass of benign Schwannoma is usually enough as treatment [17,30,41,57]. Some authors consider endoscopic enucleation a very effective and minimally aggressive technique as treatment, but it is limited to small and well-defined lesions [21]. Other therapeutic approaches include polypectomy, thoracoscopic surgery assisted by video or by a robot and surgical resection with wide margins (total, subtotal or partial esophagectomy) [6,20,58]. Video-assisted thoracoscopic surgery (VAST) has become popular because it offers a shorter postoperative period and it is less painful in comparison to thoracotomy. Robot-assisted thoracoscopic surgery (RATS) has shown greater advantages over conventional thoracoscopic approaches, such as swiveling mobility of instruments, three-dimensional vision and ergonomic comfort for the surgeon [57,73]. Regarding treatment in the literature review, we observed that the VAST (26.3%) is the most performed, followed by right posterolateral thoracotomy (TOR) (21.0%) and RATS (2.36%). The most used surgical procedure was enucleation (59.2%), followed by subtotal esophagectomy (7.4%) and endoscopic removal (5.5%) (Table 4).

The prognosis is determined by the degree of mitosis and the size of the tumor. Thus, the lower degree of mitosis and the smaller tumor, the better the prognosis. The prognosis and predictive factors for schwannoma are good since schwannoma is most often a benign tumor with a very low recurrence potential. Malignant transformation is exceptional and rare, and schwannomas tend to recur after incomplete removal^61^.

## 6 Conclusion

We reported an esophageal Schwannoma case, essentially benign, positive for S-100 protein and KI67 positive in 5% of tumor cells, which is part of the differential diagnosis of mesenchymal tumors. The tumor was treated with resection-surgical margins. The literature review shows the relevant statistical data of 54 patients with at least S-100 protein immunohistochemical marking (Table 2) and data from 9 patients with malignant Schwannoma. Therefore, we determined that women are the most affected and the tumor is essentially benign in most cases. On average, the tumor mass appears at 53.72 years old and the most prevalent symptoms are dysphagia and dyspnea. Schwannomas present negativity for SMA, Desmin, CD34, CD117 and positivity for S-100 protein and NSE. The most chosen treatment still remains the enucleation of the tumor mass.

## Conflicts of interest

The author(s) declared no potential conflicts of interest with respect to the research, authorship, and/or publication of this article.

## Sources of funding

The author(s) received no financial support for the research, authorship, and/or publication of this article.

## Ethical approval

The case report was submitted to the National Council of Health - National Commission of Ethics in Research of Brazil - ***CONEP*** and to the Research Ethics Committee of the Base Hospital Institute of the Federal District - ***IHBDF***, with registration **CAAE:** 00,711,718. 00711718.0.0000.8153

## Consent

Written informed consent was obtained from the wife of the patient for publication of this case report and accompanying images. A copy of the written consent is available for review by the Editor-in-Chief of this journal on request.

## Author contribution

Luiz Carlos de Araújo Souza was assigned to formal data analysis and statistical elaboration; Thiago David Alves Pinto and Hugo Oliveira de Figueiredo Cavalcanti were responsible for the methodology used in the pathological diagnosis; Alexandre Rezende Rezende was responsible for project management; Thiago David Alves Pinto and Luiz Carlos de Araújo Souza were responsible for the review and validation of the work; Ana Luiza Alves Nicoletti, Cinthia Mares Leão and Vinícius Carvalhêdo Cunha were responsible for the papers, writing and writing of the work.

## Registration of research studies

NA.

## Guarantor

Luiz Carlos de Araújo Souza.

## Provenance and peer review

Not commissioned, externally peer-reviewed.
