# Supplementary material for: Esophageal schwannoma: Case report and epidemiological, clinical, surgical and immunopathological analysis
Source: Int J Surg Case Rep. 2019 Jan 10;55:69–75. doi: 10.1016/j.ijscr.2018.10.084 (PMC6357786; doi:10.1016/j.ijscr.2018.10.084)
Supplement: Supplementary file 1 [file mmc1.docx]

**
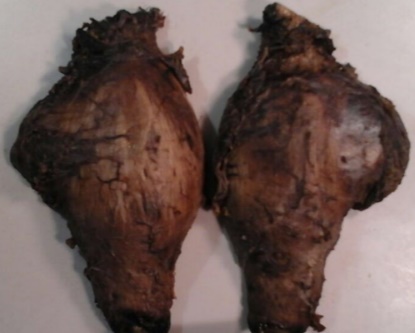
**

**
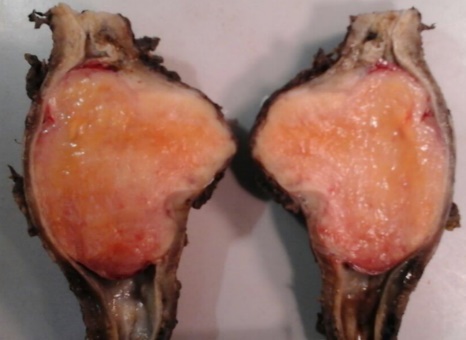
**

**Figures supplemental data 1**. Anatomical parts of the esophageal tumor after right-sided posterolateral thoracotomy with esophagectomy.
