# Supplementary material for: Esophageal schwannoma: Case report and epidemiological, clinical, surgical and immunopathological analysis
Source: Int J Surg Case Rep. 2019 Jan 10;55:69–75. doi: 10.1016/j.ijscr.2018.10.084 (PMC6357786; doi:10.1016/j.ijscr.2018.10.084)
Supplement: Supplementary file 2 [file mmc2.docx]

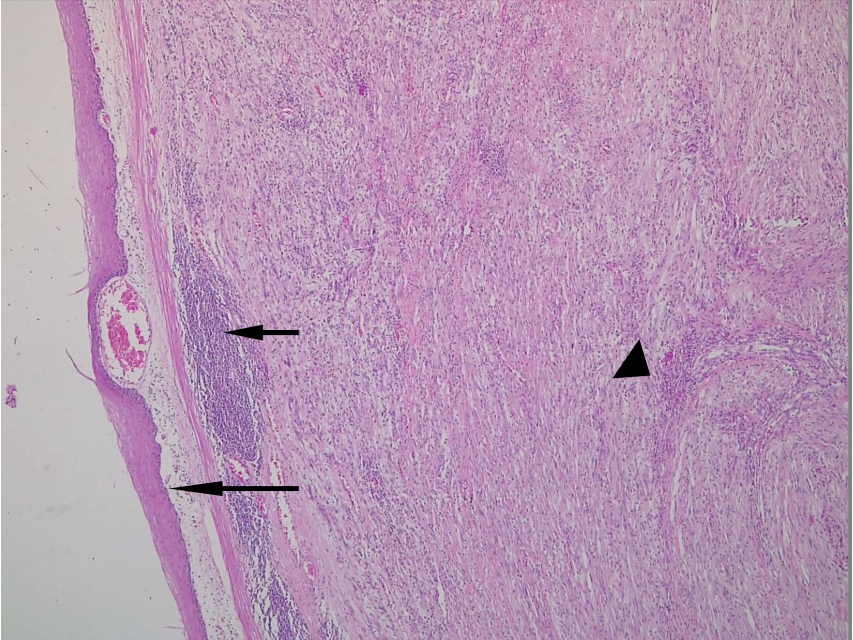


**Figures supplemental data 2.** Low grade fasciculate lesion with intralesional lymphoid aggregates (**arrowhead**) and perilesional (**short arrow**), lined by esophageal squamous mucosa (**long arrow**). (**HE 40x**)
