# Supplementary material for: Esophageal schwannoma: Case report and epidemiological, clinical, surgical and immunopathological analysis
Source: Int J Surg Case Rep. 2019 Jan 10;55:69–75. doi: 10.1016/j.ijscr.2018.10.084 (PMC6357786; doi:10.1016/j.ijscr.2018.10.084)
Supplement: Supplementary file 3 [file mmc3.docx]

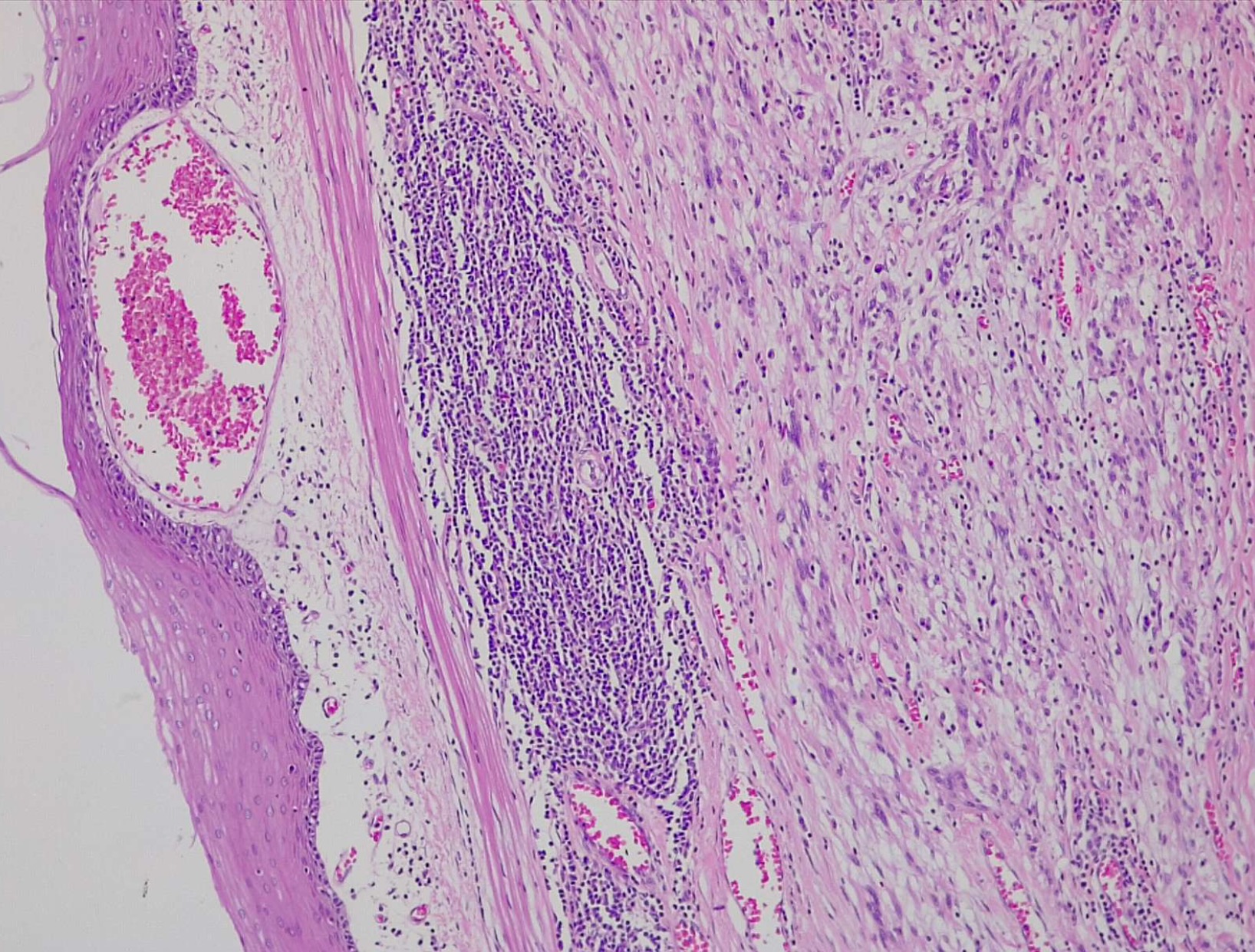


**Figures supplemental data 3.** Transition between lesion / lymphoid aggregate and esophageal squamous mucosa. (**HE 100x**)
