# Supplementary figures and images for: Esophageal schwannoma: Case report and epidemiological, clinical, surgical and immunopathological analysis
Source: Int J Surg Case Rep. 2019 Jan 10;55:69–75. doi: 10.1016/j.ijscr.2018.10.084 (PMC6357786; doi:10.1016/j.ijscr.2018.10.084)

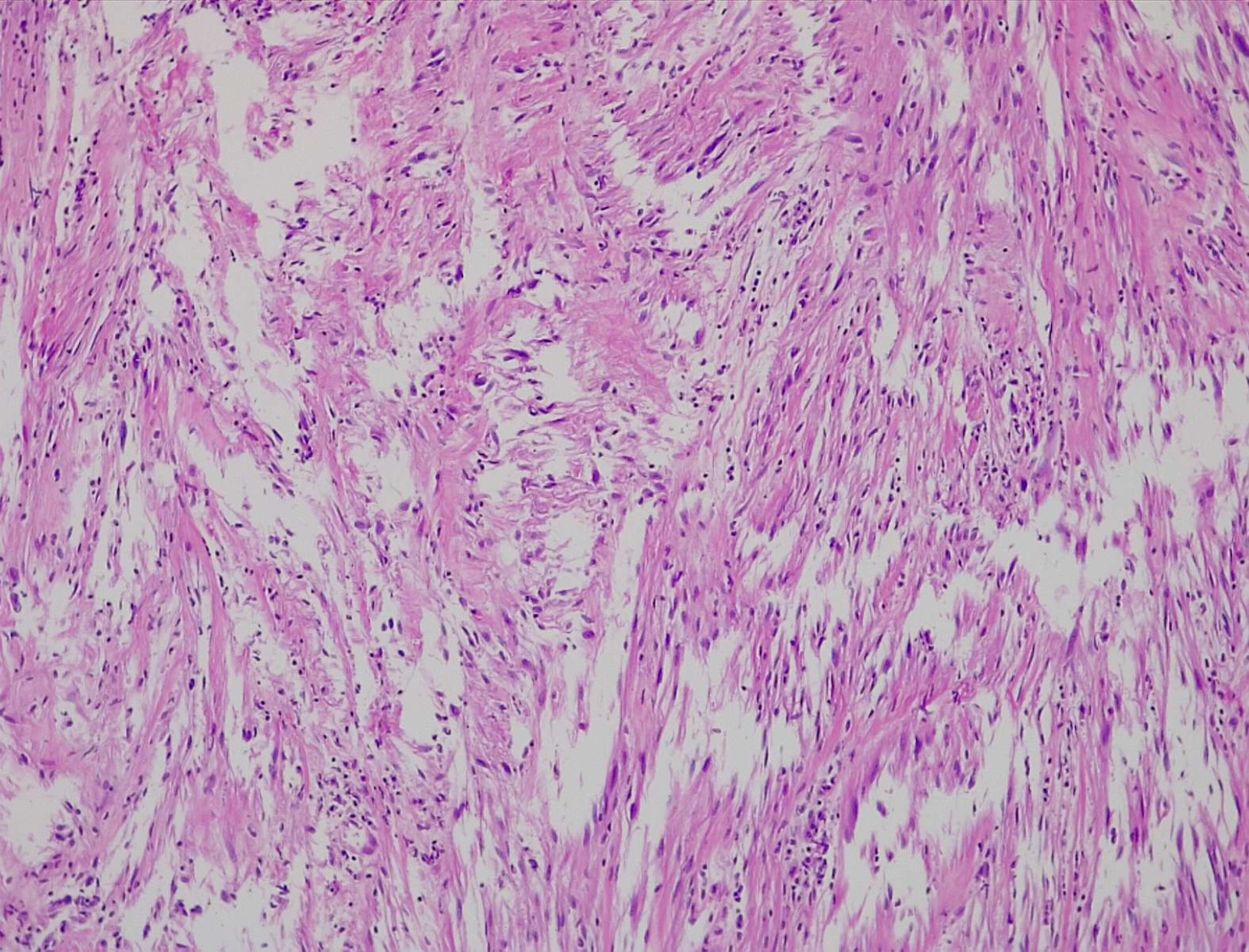


**Figures supplemental data 4.** Fascicles of the lesion sketching palisade. (**HE 100x**)

Supplement: Supplementary file 4 [file mmc4.docx]

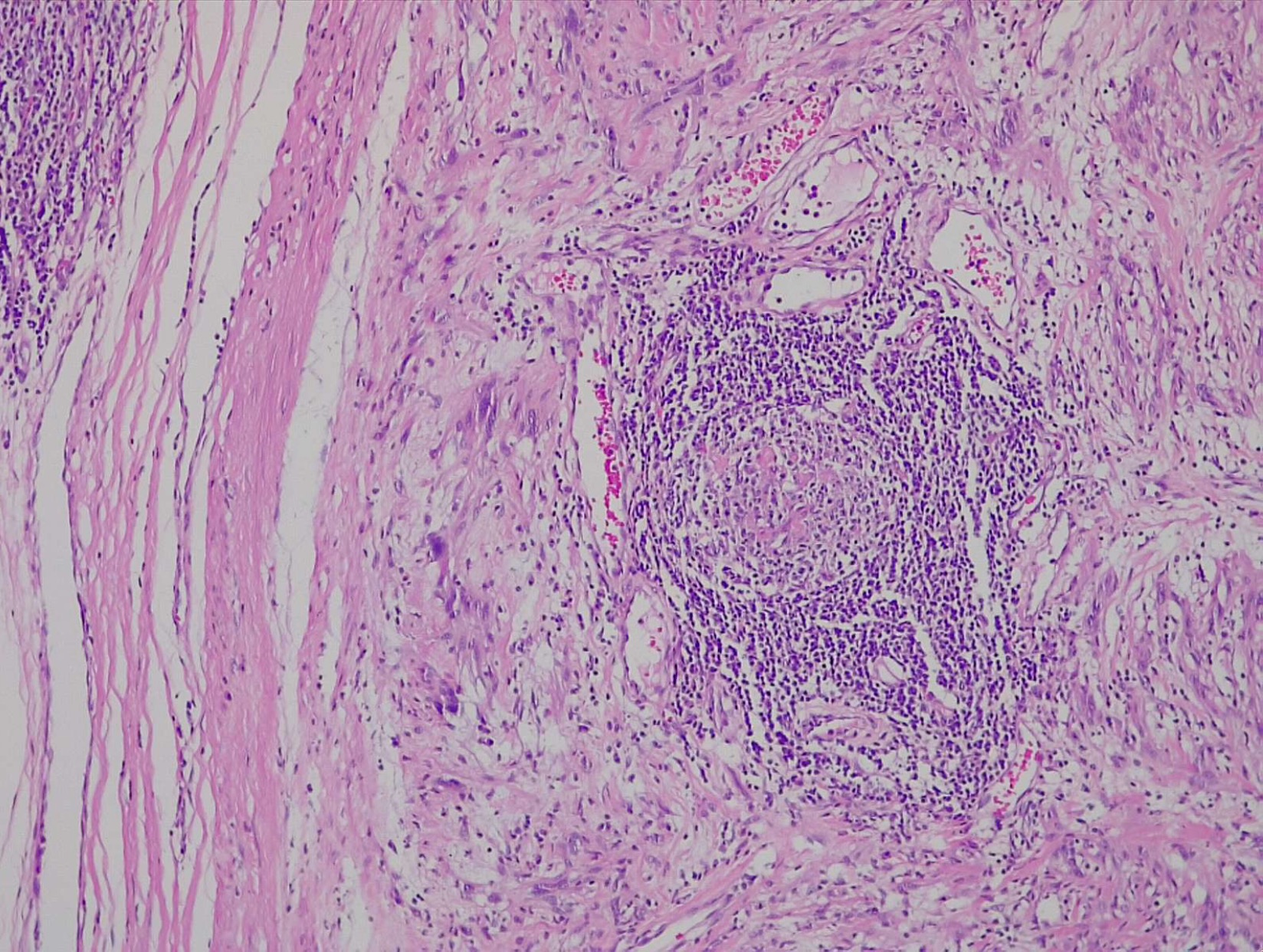


**Figures supplemental data 7.** Lymphoid follicle at the periphery of the lesion. (**HE 100x**)

Supplement: Supplementary file 7 [file mmc7.docx]

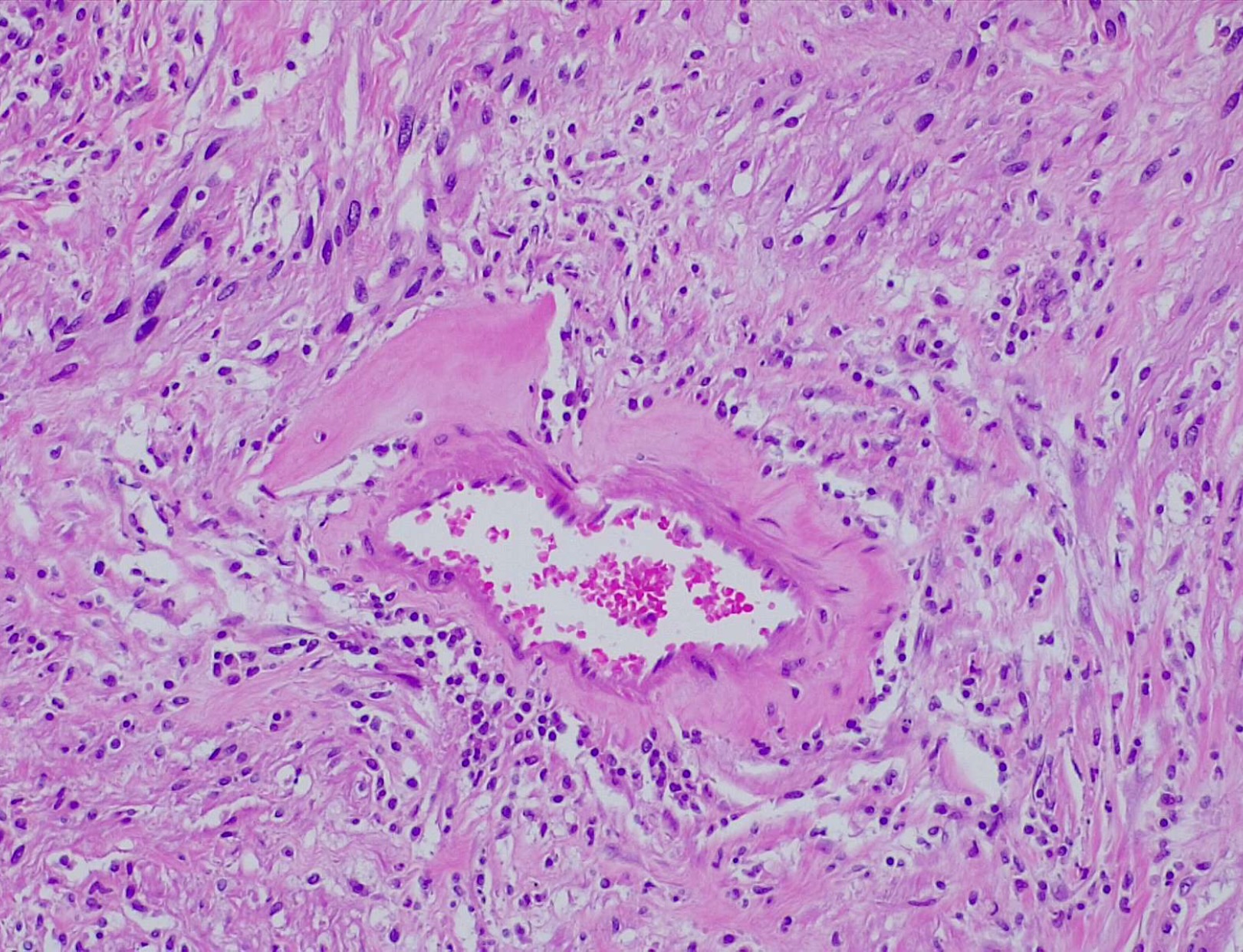


**Figures supplemental data 8.** Vessel with hyalinized wall inside the lesion. (**HE 200x**)

Supplement: Supplementary file 8 [file mmc8.docx]

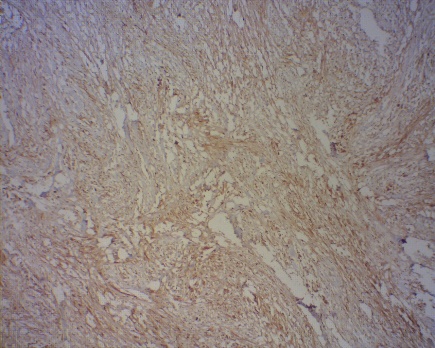


**Figures supplemental data 9**. Immunohistochemistry using Desmin antibody. (**IHQ 40x**)

Supplement: Supplementary file 9 [file mmc9.docx]
