# Supplementary material for: Esophageal schwannoma: Case report and epidemiological, clinical, surgical and immunopathological analysis
Source: Int J Surg Case Rep. 2019 Jan 10;55:69–75. doi: 10.1016/j.ijscr.2018.10.084 (PMC6357786; doi:10.1016/j.ijscr.2018.10.084)
Supplement: Supplementary file 5 [file mmc5.docx]

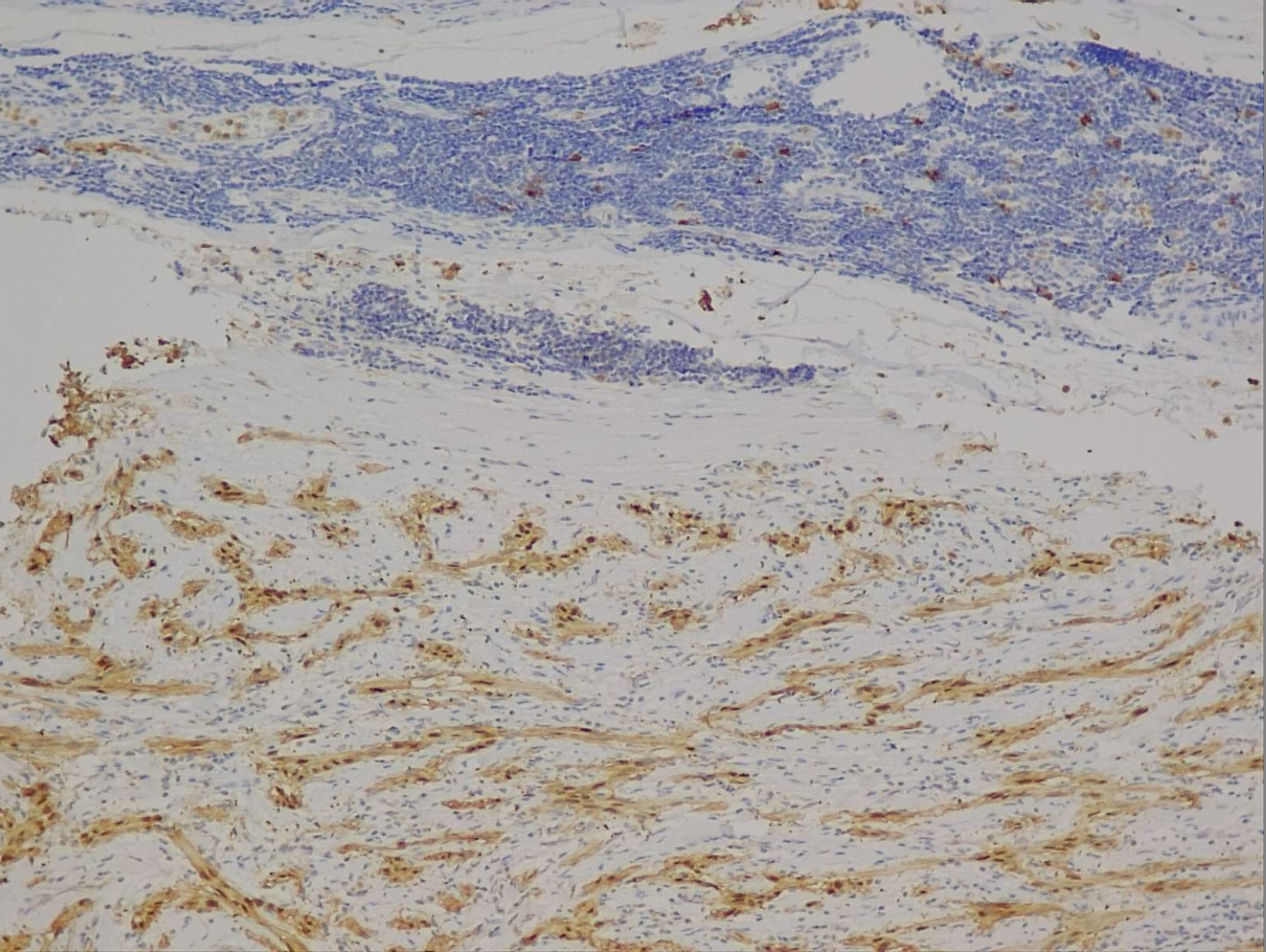


**Figures supplemental data 5.** Positive reaction for S-100 in neoplastic cells l and negative in lymphoid aggregates. (**IHQ 100x**)
