# Supplementary material for: Esophageal schwannoma: Case report and epidemiological, clinical, surgical and immunopathological analysis
Source: Int J Surg Case Rep. 2019 Jan 10;55:69–75. doi: 10.1016/j.ijscr.2018.10.084 (PMC6357786; doi:10.1016/j.ijscr.2018.10.084)
Supplement: Supplementary file 6 [file mmc6.docx]

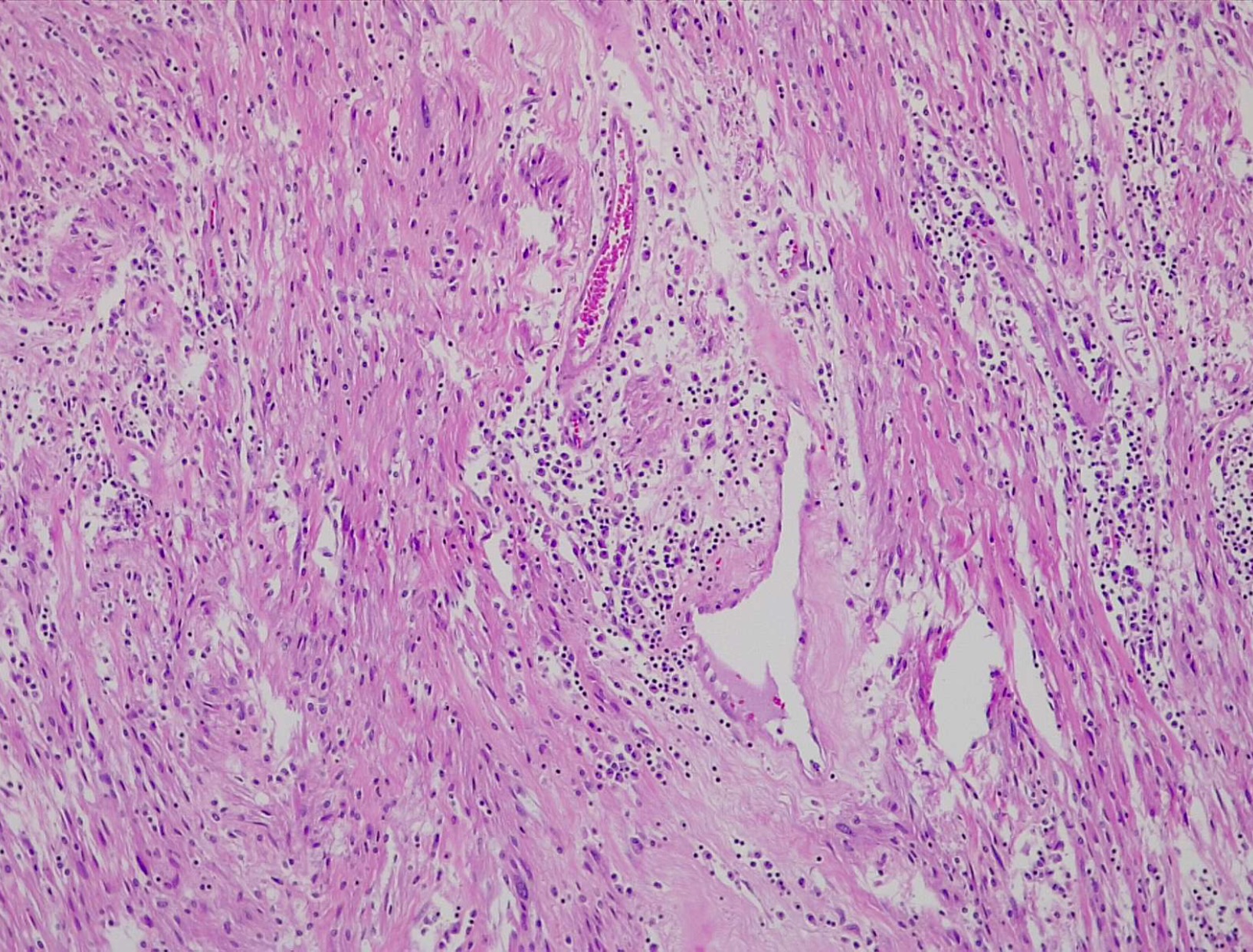


**Figures supplemental data 6.** Hyaline vessel and perivascular mononuclear infiltrate within the lesion. (**HE 100x)**
